# SARS-CoV-2 genome-wide T cell epitope mapping reveals immunodominance and substantial CD8^+^ T cell activation in COVID-19 patients

**DOI:** 10.1126/sciimmunol.abf7550

**Published:** 2021-04-14

**Authors:** Sunil Kumar Saini, Ditte Stampe Hersby, Tripti Tamhane, Helle Rus Povlsen, Susana Patricia Amaya Hernandez, Morten Nielsen, Anne Ortved Gang, Sine Reker Hadrup

**Affiliations:** 1 Department of Health Technology, Section of Experimental and Translational Immunology, Technical University of Denmark, Kongens Lyngby, Denmark.; 2 Department of Haematology, Herlev Hospital, Copenhagen University Hospital, Herlev, Denmark.; 3 Department of Health Technology, Section of Bioinformatics, Technical University of Denmark, Kongens Lyngby, Denmark.

## Abstract

T cells are important for effective viral clearance, elimination of virus-infected cells and long-term disease protection. To examine the full-spectrum of CD8^+^ T cell immunity in COVID-19, we experimentally evaluated 3141 major histocompatibility (MHC) class I-binding peptides covering the complete SARS-CoV-2 genome. Using DNA-barcoded peptide-MHC complex (pMHC) multimers combined with a T cell phenotype panel, we report a comprehensive list of 122 immunogenic and a subset of immunodominant SARS-CoV-2 T cell epitopes. Substantial CD8^+^ T cell recognition was observed in COVID-19 patients, with up to 27% of all CD8^+^ lymphocytes interacting with SARS-CoV-2-derived epitopes. Most immunogenic regions were derived from open reading frame (ORF) 1 and ORF3, with ORF1 containing most of the immunodominant epitopes. CD8^+^ T cell recognition of lower affinity was also observed in healthy donors toward SARS-CoV-2-derived epitopes. This pre-existing T cell recognition signature was partially overlapping with the epitope landscape observed in COVID-19 patients and may drive the further expansion of T cell responses to SARS-CoV-2 infection. Importantly the phenotype of the SARS-CoV-2-specific CD8^+^ T cells, revealed a strong T cell activation in COVID-19 patients, while minimal T cell activation was seen in healthy individuals. We found that patients with severe disease displayed significantly larger SARS-CoV-2-specific T cell populations compared to patients with mild diseases and these T cells displayed a robust activation profile. These results further our understanding of T cell immunity to SARS-CoV-2 infection and hypothesize that strong antigen-specific T cell responses are associated with different disease outcomes.

## Teaser

CD8^+^ T cell recognition is detected throughout the SARS-CoV-2 genome and is associated with COVID-19 disease severity.

## INTRODUCTION

The COVID-19 (Coronavirus disease 2019) pandemic caused by the highly infectious SARS-CoV-2 (severe acute respiratory syndrome coronavirus 2) has challenged public health at an unprecedented scale, causing the death of more than two million people worldwide so far (*1*). T cells perform essential functions in the control and elimination of viral infections; CD8^+^ T cells are critical for efficient clearance of virus-infected cells, whereas CD4^+^ T cells are important for supporting both the CD8^+^ T cell response and B cell-mediated production of specific antibodies. Characteristics from the ongoing pandemic suggest that T cell recognition will be critical to mediate long-term protection against SARS-CoV-2 (*2*), as the antibody-mediated response seems to decline in follow-up evaluation of convalescent patients, although its not yet understood how this affects the risk of re-infection and what antibody levels are required for disease protection (*3****5*). Furthermore, studies of the closely related SARS-CoV infection shows persistent memory CD8^+^ T cell responses even after 11 years in SARS recovered patients without B cell responses (*6*, *7*), emphasizing the potential role of CD8^+^ memory T cells in long-term protection from coronaviruses.

Several recent studies have reported robust T cell immunity in SARS-CoV-2-infected patients (*8****10*), and unexposed healthy individuals also showed functional T cell reactivity restricted to SARS-CoV-2 (*9*, *11****15*). The observed T cell cross-reactivity is hypothesized to derive from routine exposure to common cold coronaviruses (HCoV) (HCoV-OC43, HCoV-HKU1, HCoV-NL63 and HCoV-229E) that widely circulate, with 90% of the human population being seropositive for these viruses (*16*, *17*) and substantial sequence homology to the SARS-CoV-2 genome (*18*, *19*). However, the influence of such pre-existing immunity to the T cell recognition associated with COVID-19 disease is poorly understood.

SARS-CoV-2 infection can result in mild to severe disease (including death), but also a large number of asymptomatic infections are described (*20****22*). The presence of pre-existing T cell immunity, represented by cross-reactive T cells, could have strong implications for how individuals respond to SARS-CoV-2 infection. However, their biological role upon encounter with SARS-CoV-2 infection remains unclear, and their contribution to disease protection needs to be determined. Furthermore, in severe clinical disease, cytokine release syndrome is reported, and might in some cases, be dampened by immunosuppressive medication or anti-IL6 antibody therapy (*23*, *24*). Such clinical characteristics point to a potential uncontrolled immune response with the involvement of strong T cell activation.

CD8^+^ T cells are activated by a specific interaction between the T cell receptor (TCR) and peptide-antigen presented by major histocompatibility complex I (MHC-I) molecules on the surface of virus-infected cells. Although SARS-CoV-2-specific immunity has been reported both in the context of COVID-19 and pre-existing T cells, the full spectrum of exact antigens (minimal peptide epitope) within the viral genome, associated with this immunity and their immunodominance in SARS-CoV-2-infected patients is not fully described. Using our large-scale T cell detection technology based on DNA-barcoded peptide-MHC multimers (*25*), we have mapped T cell recognition throughout the complete SARS-CoV-2 genome, identified the exact epitopes recognized by SARS-CoV-2-specific CD8^+^ T cells, and characterized immunodominance of these epitopes in COVID-19 disease. Broad T cell recognition toward SARS-CoV-2-derived peptides was also identified in SARS-CoV-2 unexposed healthy individuals, with a large overlap in the peptide-MHC complexes recognized in the two groups. However, T cell recognition was substantially enhanced in the patient group, with SARS-CoV-2-reactive T cells accounting for up to 27% of all CD8^+^ T cells. Furthermore, we have evaluated the phenotypic characteristics of SARS-CoV-2-specific T cells and correlated their activation signatures with disease severity.

## RESULTS

### SARS-CoV-2-specific CD8***^+^***T cells recognize a broad range of epitopes

**Fig. 1:**
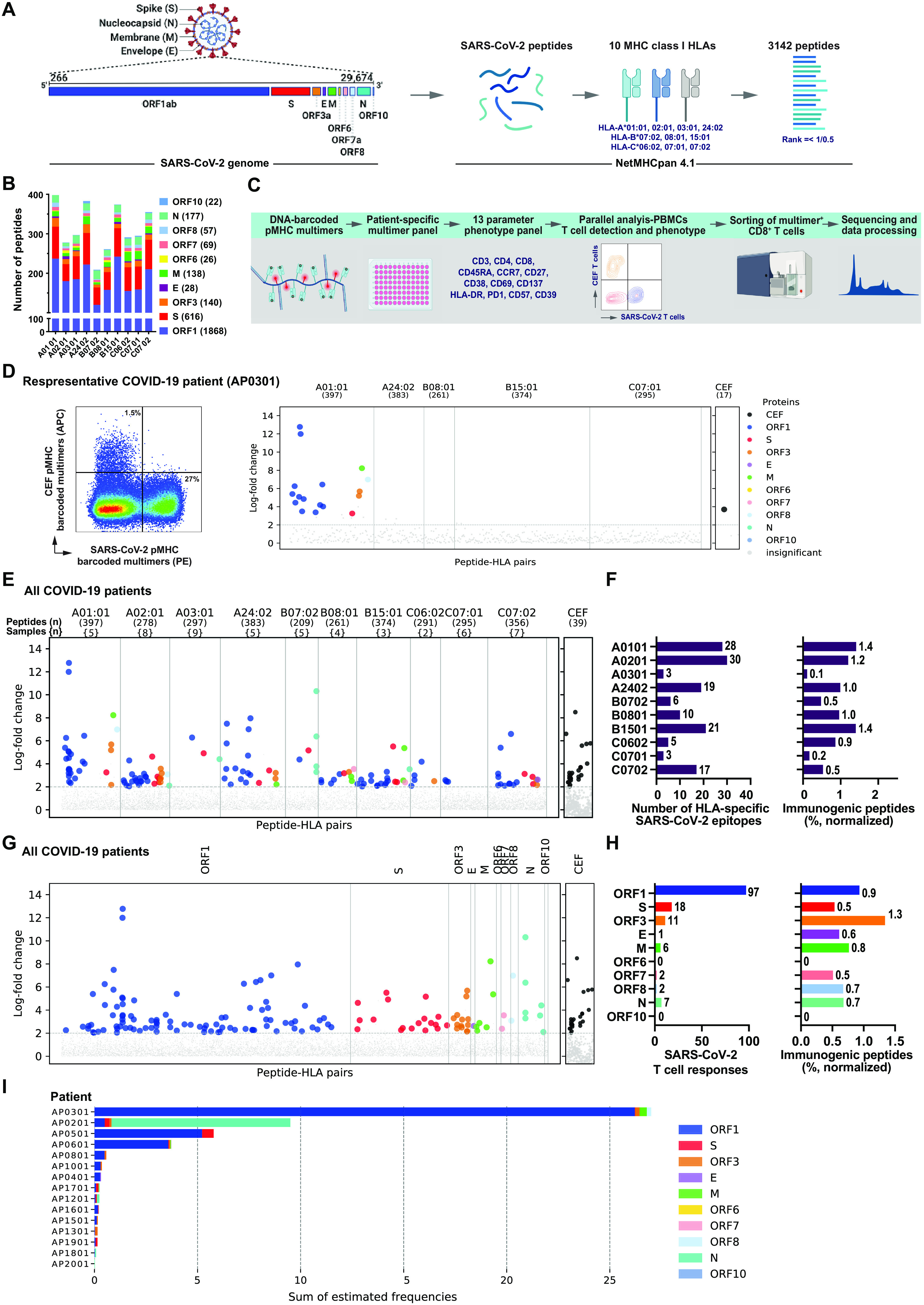
CD8^+^ T cell epitope mapping in SARS-CoV-2. (**A**) Schematic representation of the complete SARS-CoV-2 genome used for the identification of 3141 peptides with predicted binding rank (NetMHCpan 4.1) of 0.5 (ORF1 protein) and 1 (all remaining proteins) for ten prevalent HLA-A, B, and C molecules. (**B**) Bar plot showing the distribution of SARS-CoV-2 peptides related to their HLA-restriction (3141 peptide-HLA pairs) across the viral genome. Total peptide-MHC specificities analyzed for each protein are shown in parentheses next to the respective SARS-CoV-2 protein. (**C**) Experimental pipeline to analyze T cell recognition toward the SARS-CoV-2-derived HLA-binding peptides in PBMCs using DNA-barcoded peptide-MHC (pMHC) multimers. A 13-antibody panel was used for phenotype analysis of pMHC multimer positive CD8^+^ T cells. pMHC multimers binding CD8^+^ T cells were sorted based on PE (SARS-CoV-2-specific) or APC (CEF-specific) signal and sequenced to identify antigen-specific CD8^+^ T cells. (**D**) Representative analyses for SARS-CoV-2-restricted T cell populations in a COVID-19 patient. **Left**, flow cytometry plot of pMHC multimer staining of CD8^+^ T cells from a COVID patient stained with pMHC multimer panel showing SARS-CoV-2 (PE) and CEF (APC) multimer^+^ T cells that were sorted for DNA-barcode analysis to identify epitopes recognition. **Right**, CD8^+^ T cell recognition to individual epitopes were identified based on the enrichment of DNA barcodes associated with each of the tested peptide specificities (LogFc>2 and p <0.001, using *barracoda*). Significant T cell recognition of individual peptide sequences are colored based on their protein of origin and segregated based on their HLA-specificity. The black dot show CD8^+^ T cells reactive to one of the CEF peptides (here, CMV pp65; YSEHPTFTSQY-HLA-A01:01). All peptides with no significant enrichments are shown as gray dots. (**E**) Summary of all the T cell recognition to SARS-CoV-2-derived peptides identified in the 18 analyzed COVID-19- patients. In parentheses, number of peptides tested for each HLA (upper row) and the number of patients analyzed for each HLA pool (lower row). Each dot represents one peptide-HLA combination per patient, and are colored according to their origin of protein, similar to shown in panel A. The black dots show CD8^+^ T cells reactive to the CEF peptides in all analyzed patients. (**F**) Bar plots summarize the number of HLA-specific SARS-CoV-2 epitopes identified and the HLA-restricted immunogenicity (% immunogenic peptides) in the analyzed patient cohort. Immunogenicity represents the fraction of T cell recognized peptides out of the total number of peptides analyzed for a given HLA-restriction across the HLA-matching donors (% normalized). (**G**) Similar to E, a summary of SARS-CoV-2-specific T cell responses separated based on the protein of origin. (**H**) Bar plots show the number of epitopes derived from each of the SARS-CoV-2 protein and their immunogenicity score (% immunogenic peptides). (**I**) Estimated frequencies (% of total CD8^+^ T cells) as sum of all the SARS-CoV-2 epitope reactive T cells identified in individual COVID-19 patients. Bars are colored according to the protein origin of the recognized epitopes.

To reveal the full spectrum of T cell immunity in COVID-19 disease, we used a complete SARS-CoV-2 genome sequence (*26*) to identify immunogenic minimal epitopes recognized by CD8^+^ T cells. Using NetMHCpan 4.1 (*27*), we selected 2204 potential HLA binding peptides (911 amino acids) for experimental evaluation. These peptides were predicted to bind one or more of ten prevalent MHC-I molecules, including HLA-A (A01:01, A02:01, A03:01, and A24:02), -B (B07:02, B08:01, B15:01), and -C (C06:02, C07:01, and C07:02) loci, leading to a total 3141 peptide-MHC specificities for experimental evaluation (**Fig. 1A****, Table S1**). Epitope predictions is covering the full viral genome, with ORF1 being the largest gene region and hence including the highest number of predicted peptides (**Fig. 1B**). T cell reactivity toward these peptides was analyzed for 18 COVID-19 patients. In this cohort, 11 patients had severe disease requiring hospital care and seven patients had mild disease not requiring hospitalization. Blood samples were collected during the active phase of the infection, as close as possible after the first positive SARS-CoV-2 test (**Table S2**). The mean HLA coverage that could be obtained using the ten selected MHC-I molecules was 3.1 HLA per patient and patients were evaluated using on average 972 DNA-barcoded peptide-MHC (pMHC) multimers per patient (**Fig. S1A**) (*25*). Briefly, each pMHC complex is multimerized on a PE- (Phycoerythrin) or APC- (Allophycocyanin) labeled dextran backbone and tagged with a unique DNA-barcode. DNA-barcoded pMHC multimers are then pooled to generate an HLA matching patient-tailored pMHC multimer panel, which is incubated with patient-derived PBMCs (peripheral blood mononuclear cells), and multimers bound to CD8^+^ T cells are sorted and sequenced to identify T cell recognition toward the probed pMHC complexes. For comparative evaluation, we also included 39 T cell epitopes from common viruses: cytomegalovirus (CMV), Epstein-Barr virus (**E**BV), and influenza (**F**lu) virus (CEF) (**Table S3**, **Fig. 1C**).

We found broad and strong SARS-CoV-2-specific CD8^+^ T cell responses in COVID-19 patients, contributing up to 27% of the total CD8^+^ T cells (**Fig. 1D**). A substantial selection of T cells specific to individual immunogenic epitopes measuring up to 14% of the total T cells was detected in several patients (**Fig. 1D****, Fig. S2,** and **Table S4**). In total, we identified T cell responses to 142 pMHC complexes corresponding to 122 unique SARS-CoV-2 T cell epitopes across the ten analyzed HLAs (**Fig. 1E**) dominated by peptides with high-affinity binding to their corresponding HLA molecule (**Fig. S1B**). We also detected 25 T cell responses to CEF-derived peptides across the 18 COVID-19 patients (**Fig. 1E** and **Table S5**). For the SARS-CoV-2-derived peptides, HLA-A01:01, A02:01, and B15:01 presentation dominated in terms of the total number of identified epitopes as well as the immunogenicity score (i.e., the number of T cell responses normalized to the number of probing pMHC multimers and the number of patients analyzed) (**Fig. 1F**). HLA-A03:01 and C07:01 specific peptides showed the least T cell reactivity (three epitopes each) despite being analyzed in nine and six patients, respectively (**Fig. 1E**). Most of the immunogenic epitopes were mapped to the ORF1 protein, followed by S and ORF3 proteins (**Fig. 1, G and H**, and **Table S4**). Given the size difference of the viral proteins, the immunogenicity score was used to evaluate their relative contribution to T cell recognition. Based on such evaluation, we observe that peptides derived from ORF3 displayed the highest relative immunogenicity (in terms of T cell recognition), followed by ORF1 protein (**Fig. 1H**). The overall frequency of SARS-CoV-2-reactive T cells (the sum of estimated frequencies for all SARS-CoV-2-specific T cells) in individual COVID-19 patients showed a broad range of T cell involvement and variation in terms of T cell recognition to individual SARS-CoV-2 proteins (**Fig. 1I**).

In summary, we report SARS-CoV-2-specific CD8^+^ T cell immunity toward several epitopes and substantially high presence of SARS-CoV-2-specific T cells in several COVID-19 patients. The ORF1 protein contributes the most to T cell recognition of SARS-CoV-2, but is also by far the largest group of proteins. When protein size is considered, ORF3 and ORF1, are the viral regions most frequently recognized by CD8^+^ T cells.

### Strong immunodominance of SARS-CoV-2 derived peptides in COVID-19 patients.

**Fig. 2:**
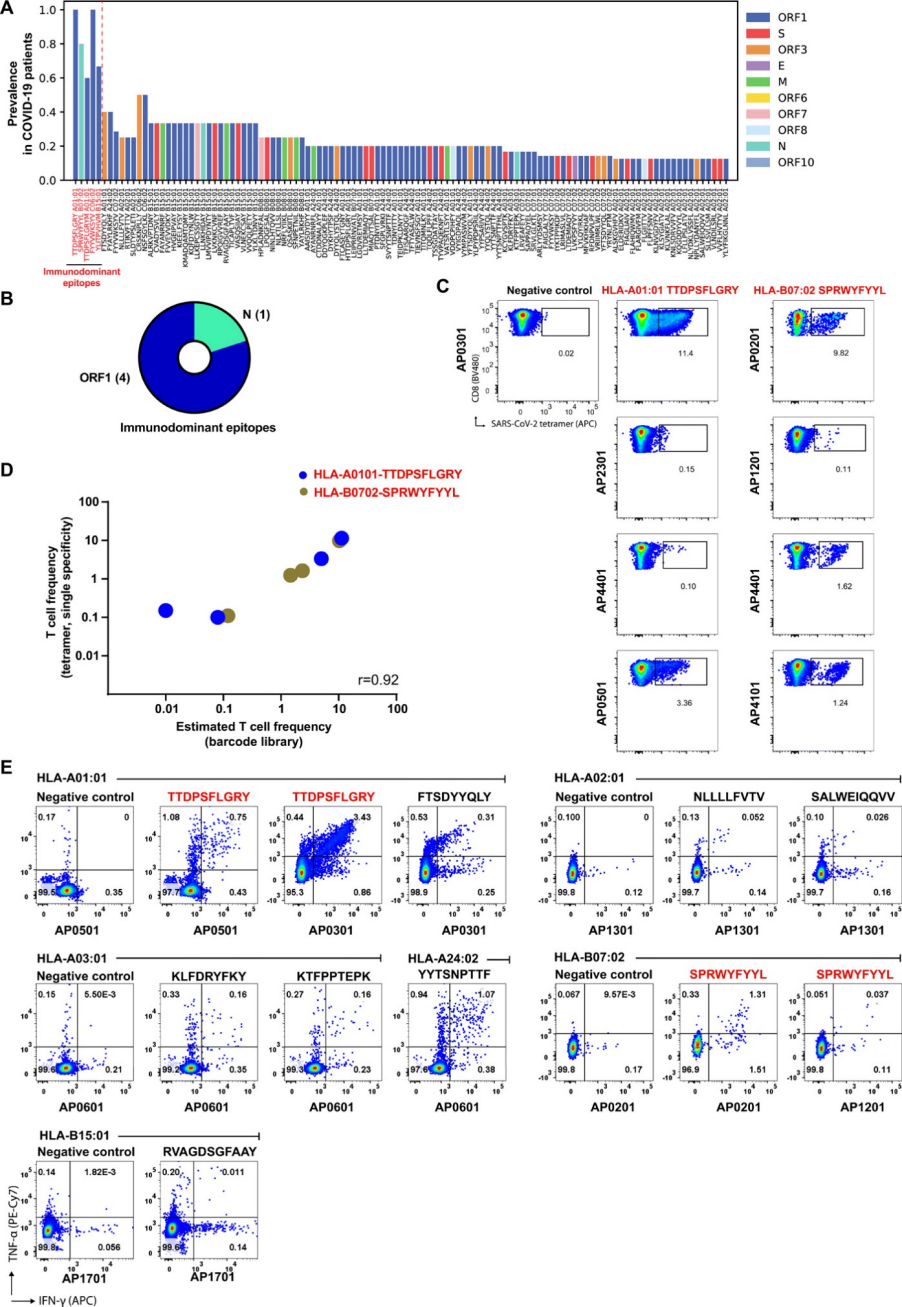
Strong immunodominance of SARS-CoV-2 derived peptides in COVID-19 patients. (**A**) The prevalence of T cell recognition toward the individual epitopes detected in COVID-19 patients. Dotted line indicates the epitopes determined as immunodominant, based on the presence of T cell recognition in more than 50% analyzed patients (marked in red throughout this figure). Bars are colored according to their protein of origin, similar as shown in Fig. 1. (**B**) Pie chart of immunodominant epitopes distributed according to their protein of origin. (**C**) APC labeled pMHC tetramer-based analyses of CD8^+^ T cells in PBMCs of seven COVID-19 patients recognizing A01:01/TTDPSFLGRY or B07:02/SPRWYFYYL immunodominant epitopes. Gated population shows the percentage of T cells recognizing pMHC tetramers out of total CD8^+^ T cells. (**D**) Correlation of estimated T cell frequencies determined using DNA-barcoded multimer estimation (**Table S4**) and using conventional tetramer analysis (panel C) for the two most immunodominant epitopes in seven COVID-19 patients. Spearman correlation (p =0.002, r =0.92). (**E**) Functional validation of SARS-CoV-2-specific T cell responses identified in COVID-19 patient samples. Flow cytometry plots showing intracellular cytokine staining of PBMCs from COVID-19 patients pulsed with selected SARS-CoV-2-derived peptides, selected based on the CD8^+^ T cell responses identified from the DNA-barcoded multimer analysis (**Table S4**). PBMCs from seven COVID-19 patients were analyzed for functional activation after stimulation with indicated peptides or with an HLA-matching irrelevant peptide (negative control). The numbers on the plot indicate the frequency (%) of CD8^+^ T cells positive for the analyzed cytokines, IFN- and TNF-. The gating strategy of the flow cytometry analysis is shown in Fig. S5A.

Of the 122 epitopes recognized by T cells in the patient cohort, five were determined as immunodominant based on the presence of T cell recognition in >50% in the tested samples with the given HLA molecule, and T detection identified in at least two or more patients (**Fig. 2A**). Surprisingly, in our patient cohort, none of the immunodominant epitopes were derived from the S protein, despite this being the second-largest protein (**Fig. 2B**). Among the immunodominant epitopes, a very robust HLA-associated immunodominance was observed for two of the epitopes: HLA-A01:01-TTDPSFLGRY-specific (and its variant peptides TTDPSFLGRYM and HTTDPSFLGRY), with specific T cells detected in all five analyzed patients (estimated frequency reaching up to 25% of total CD8^+^ T cells); and HLA-B07:02-SPRWYFYYL, with specific T cells observed in four of the five patients evaluated (estimated frequency up to 10%) (**Fig. 2A****, Table S4**). To validate the T cell responses identified for the two most immunodominant epitopes (TTDPSFLGRY, and SPRWYFYYL), we determined the presence of these T cells using conventional fluorophore-labeled pMHC tetramers in seven COVID-19 patients. For both immunodominant epitopes, the frequency of T cells determined by the individually-labeled pMHC tetramers correlated to the frequencies determined based on the DNA-barcode labeled MHC multimer reagents (at a range from 0.01% to 11% of the total CD8^+^ T cells) (**Fig. 2, C and D**). Next, we evaluated the cytokine secretion capacity of the SARS-CoV-2 specific T cells by stimulating PBMCs (same timepoint as used for T cell identification) with respective epitopes. SARS-CoV-2 peptide-induced secretion of IFN- and TNF- was detected in all seven patients, confirming functional activation of T cells raised against dominant as well as non-dominant epitope (**Fig. 2E****, Table S6**).

### Low-avidity recognition toward SARS-CoV-2-derived peptides in healthy individuals

**Fig. 3:**
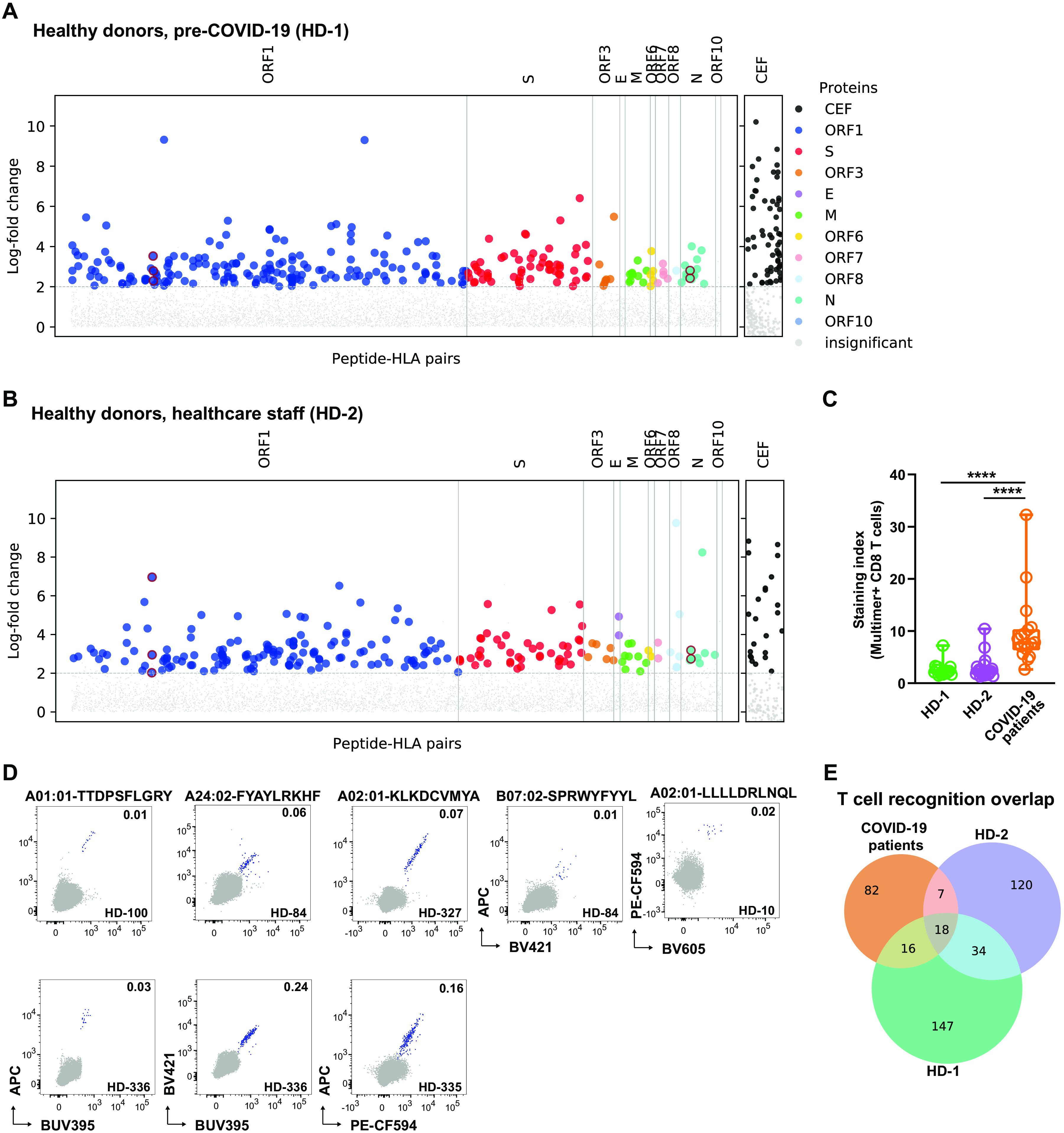
Broad reactivity toward SARS-CoV-2-derived peptides in healthy individuals. (**A**) CD8^+^ T cell recognition to individual SARS-CoV-2-derived peptides (**Table S7**) and CEF peptides (**Table S5**) in the pre-COVID-19 healthy donor cohort (n=18 individuals) identified based on the enrichment of DNA barcodes associated with each of the tested peptide specificities (LogFc>2 and p < 0.001, *barracoda*). Significant SARS-CoV-2-specific T cell recognition of individual peptide sequences are colored and segregated based on their protein of origin. The black dots show CD8^+^ T cells reactive to the CEF peptides in all analyzed donors. (**B**) T cell recognition in the high-exposure risk healthy donor cohort (**Tables S7 and S5**) (n=20 individuals). (**C**) Staining index of CD8^+^ T cells binding SARS-CoV-2-specific pMHC multimers in the three evaluated cohorts. One-Way ANOVA (Kruskal-Wallis test) p <0.0001 (patient vs. HD-1 <0.0001, patient vs. HD-2 <0.0001); n=18 (patient), n=18 (HD-1), and n=20 (HD-2). (**D**) Flow cytometry dot plots showing *in-vitro* expanded T cells from healthy donors recognizing SARS-CoV-2 derived epitopes, detected by combinatorial tetramer staining. T cell binding to each pMHC specificity is detected using pMHC tetramers prepared in a two-color combination (blue dots), gray dots show tetramer negative T cells, and the number on the plots shows the frequency (%) of tetramer^+^ of the CD8^+^ T cells. Gating strategy used for the flow cytometry analysis is shown in **Fig. S11A**. (**E**) Venn diagram illustrating the overlap of T cell recognition toward SARS-CoV-2-derived peptides in COVID-19 patient and healthy donor cohorts.

In order to examine the potential for pre-existing SARS-CoV-2-reactive T cells, we next analyzed healthy individuals for T cell recognition against all 3141 SARS-CoV-2-derived peptides. We selected two healthy donor cohorts: the first cohort included SARS-CoV-2 unexposed healthy individuals (HD-1; n=18 individuals, PBMCs collected before the COVID-19 pandemic), and the second cohort included healthcare staff at high-risk of SARS-CoV-2 exposure but who did not test positive (HD2; n=20 individuals, PBMCs collected during COVID-19 pandemic). CD8^+^ T cells from SARS-CoV-2 unexposed healthy individuals showed broad-scale T cell recognition toward SARS-CoV-2-derived peptides across the whole viral genome (**Fig. 3A****, Fig. S3,** and **Table S7**). Cumulatively, 214 SARS-CoV-2-derived peptides were recognized by T cells in 16 out of the 18 analyzed samples. The high-risk COVID-19 healthy cohort showed similar T cell recognition toward 178 SARS-CoV-2 epitopes (**Fig. 3B****, Table S7**) in 15 of the 20 donors. T cell recognition in healthy donors was directed equally toward ORF1 and S proteins, whereas ORF3 derived peptides was recognized less in the healthy donor cohort compared to the COVID-19 patient cohort (**Fig. S3B**). Interestingly, the immunodominant T cell epitopes from ORF1 identified in the patient cohort was not among the most prevalent responses in the healthy donors (**Fig. S3C**).

Despite such broad T cell-recognition in both healthy donor cohorts, the presence of SARS-CoV-2 recognizing T cells seems to be of low frequency with limited separation of the CD8^+^ T cells binding to the pool of DNA-barcoded pMHC multimers (**Fig. S4A**) and measured by significantly lower staining-index of the pMHC multimer binding in healthy donors compared to patients (**Fig. 3C**). Consequently, a direct estimate of the frequency of the SARS-CoV-2 reactive T cell populations in the individual healthy donors was not feasible. The low frequency and limited separation of these T cells were confirmed by independent analysis using conventional pMHC tetramers for several individual epitopes in healthy donor PBMCs (**Fig. S4B**). Together, these data suggest a lower TCR avidity to the probed pMHC in healthy individuals compared to COVID-19 patients, which could represent potential cross-reactivity from existing T cell populations potentially raised against other coronaviruses (such as common cold viruses HCoV-HKU1, HCoV-229E, HCoV-NL63, and HCoV-OC43) that share some level of sequence homology with SARS-CoV-2, as suggested in recent reports (*13*, *17*, *19*).

To further validate the presence of low frequency T cells in healthy donors, we expanded T cells in vitro from several COVID-19-unexposed healthy donors and measured T cell binding using conventional pMHC tetramers. Based on in vitro peptide-driven expansion pMHC tetramer binding T cell populations were verified in multiple donors, recognizing SARS-CoV-2-derived peptides, including immunodominant epitopes across four HLAs (A01:01-TTDPSFLRGY, A02:01-LLLLDRLNQL, A02:01-KLKDCVMYA, A24:02-FYAYLRKHF, and B07:02-SPRWYFYYL) (**Fig. 3D**). Although these T cell responses were of low frequency, a functional cytokine response (measured by IFN- and TNF- production) was observed in in vitro expanded T cell cultures when re-stimulated with individual peptide epitopes or epitope pools (**Fig. S5**). Importantly, forty-one of the COVID-19 immunogenic peptides, including the immunodominant peptides, identified in the patient cohort were also recognized by T cells of healthy donors, this includes the two most frequently observed epitopes of SARS-CoV-2: HLA-A01:01-TTDPSFLRGY and HLA-B07:02-SPRWYFYYL (**Fig. 3E****, Table S7**). Altogether, we show a full spectrum of T cell recognition toward SARS-CoV-2-derived peptides in healthy donors; this is detected at low frequency and show characteristics of low-avidity interaction based on the staining index of the pMHC multimer interaction.

### Enhanced activation profile of SARS-CoV-2-specific T cells associated with COVID-19 disease severity

**Fig. 4:**
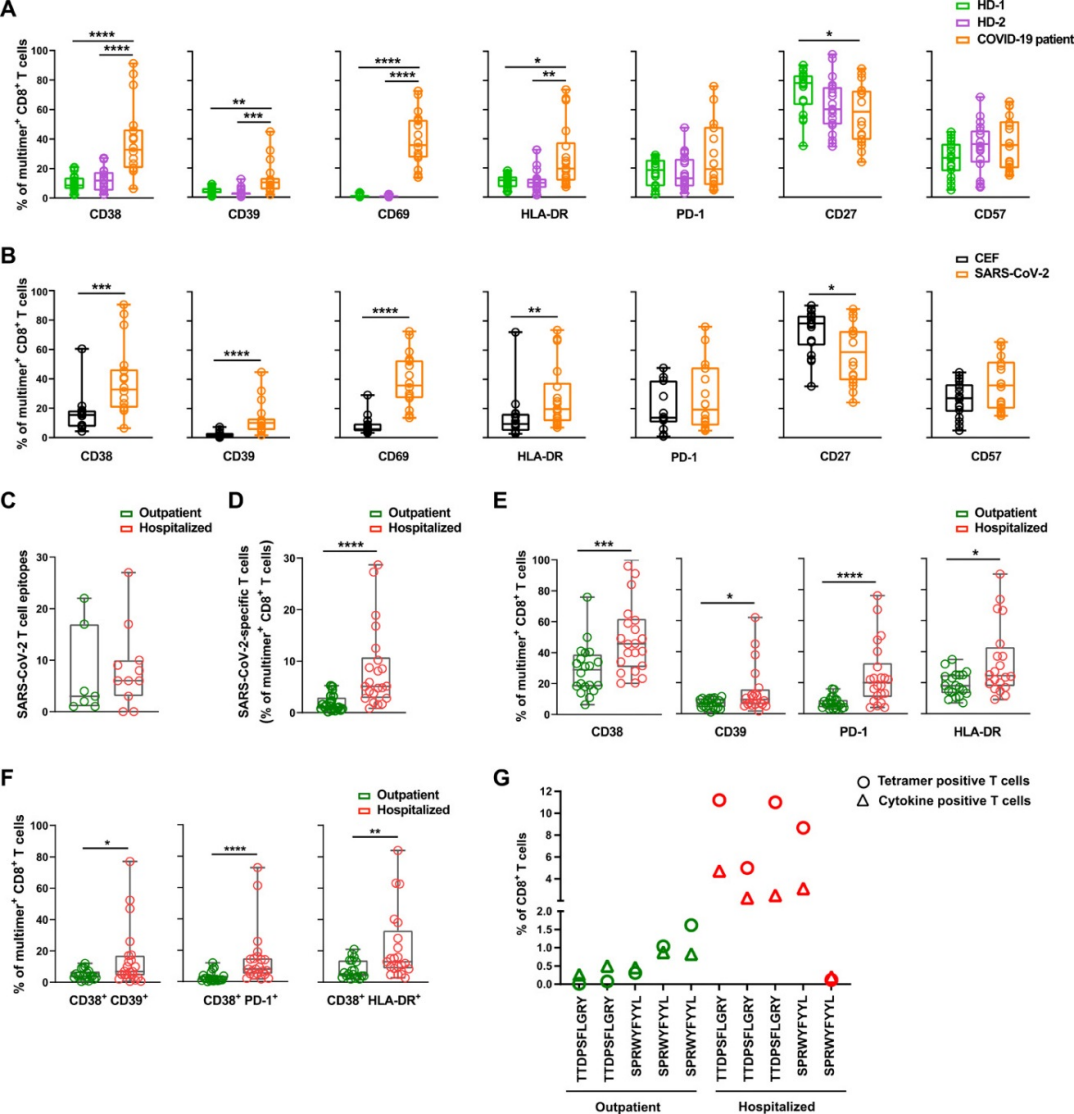
Enhanced activation profile of SARS-CoV-2-specific T cells correlates with COVID-19 disease severity. (**A**) Box plots comparing percentages of SARS-CoV-2 pMHC multimer binding CD8^+^ T cells expressing the indicated phenotype surface markers in the COVID-19 patient and the two healthy donor cohorts (n=18 individuals for each cohort). Each dot represents one sample. Frequencies were quantified from flow cytometry data processed using the gating strategy applied in **Fig. S11**. P-values for One-Way ANOVA (Kruskal-Wallis test): CD38 <0.0001 (HD-1 vs. patient <0.0001, HD-2 vs. patient <0.0001), CD39 <0.0001 (HD-1 vs. patient =0.006, HD-2 vs. patient <0.0001), CD69 <0.0001 (HD-1 vs. patient <0.0001, HD-2 vs. patient <0.0001), HLA-DR =0.002 (HD-1 vs. patient =0.02, HD-2 vs. patient <0.004), and CD27 =0.03 (HD-1 vs. patient =0.03). (**B**) Box plots comparing the percentage of SARS-CoV-2 pMHC multimer^+^ (n=18 patients) and CEF pMHC multimer^+^ (n=14 patients) CD8^+^ T cells expressing the indicated surface markers in the COVID-19 patient cohort. Each dot represents one sample. P-values for hypothesis (Mann-Whitney) test: =0.0002 (CD38), <0.0001 (CD39), 0.0001 (CD69), =0.009 (HLA-DR), =0.04 (CD27). (**C**) Number of SARS-CoV-2 epitopes recognized by T cells in outpatient (n=7) and hospitalized (n=11) patient samples. (**D**) Box plots show frequencies of SARS-CoV-2 multimer^+^ CD8^+^ T cells in outpatient (n = 20) and hospitalized patients (n=21). P-value (Mann-Whitney test) = <0.0001. (**E**) Box plots showing the percentage of SARS-CoV-2 pMHC multimer positive CD8^+^ T cells expressing the indicated surface markers in outpatients (n=20) and hospitalized patients (n=21). Each dot represents one sample. P-values for hypothesis (Mann-Whitney) test: =0.001 (CD38), =0.036 (CD39), <0.0001 (PD-1), and =0.027 (HLA-DR). (**F**) Comparison of the frequency of SARS-CoV-2 pMHC multimer+ CD8^+^ T cells expressing activation markers (CD39, and HLA-DR) and PD-1 in combination with CD38 (as shown in the representative plots (Fig. S8), in hospitalized and outpatient samples. P-values for hypothesis (Mann-Whitney) testing: =0.04 (CD38^+^ CD39^+^), = 0.005 (CD38^+^ HLA-DR^+^), and <0.0001 (CD38^+^ PD-1^+^). (**G**) Comparison of tetramer binding (conventional single-color tetramers) and functional (cytokine secreting) T cells recognizing the two immunodominant epitopes in 10 patients, grouped according to COVID-19 disease severity.

For phenotypic characterization of SARS-CoV-2-specific CD8^+^ T cells, we combined pMHC multimer analysis with a 13-parameter antibody panel (**Table S8**) and evaluated the phenotype of the SARS-CoV-2-reactive T cell populations in COVID-19 patients and healthy donors. This furthermore allowed us to evaluate whether the multimer-specific T cell profile of the high-risk for COVID-19 healthy cohort (HD-2) has any distinct features compared to the unexposed cohort (HD-1), despite both cohorts containing presumably unexposed individuals. Dimensional reduction using Uniform Manifold Approximation and Projection (UMAP) showed distinct clustering of SARS-CoV-2 multimer-reactive T cells of the COVID-19 patient cohort compared to the two healthy donor cohorts with higher expression of activation markers CD38, CD69, CD39, HLA-DR, CD57, and reduced expression of CD8 and CD27 (**Fig. S6**). Compared to both healthy donor cohorts, we observed that more SARS-CoV-2-reactive T cells from COVID-19 patients expressed the activation markers CD38, CD39, CD69 and HLA-DR, and showed a late-differentiated effector memory profile of reduced CD27 (**Fig. 4A**). We did not observe activation of SARS-CoV-2-specific multimer-positive T cells in the high-risk COVID-19 healthy cohort, except for non-significant trends for reduced CD27 and increased CD57 expression (**Fig. 4A**). SARS-CoV-2 reactive T cells in patients and healthy donor cohorts showed a similar distribution of memory subsets (determined by CCR7 and CD45RA expression); however, higher expression of T cell activation markers (**Fig. S7**) was observed in EM (effector memory) and TEMRA (terminally differentiated effector memory) subsets in patients. Furthermore, the highly activated and differentiated T cell phenotype in COVID-19 patients was distinct to SARS-CoV-2-specific T cells and not observed for CEF-specific T cells detected in the same cohort (**Fig. 4B**). We also observed no difference in CEF-specific multimer positive T cells between the three cohorts in similar analysis (**Fig. S8A**). Additionally, we compared the expression of T cell activation markers in combination with the inflammatory response marker CD38 on multimer-positive CD8^+^ T cells across the three cohorts, which showed significantly enhanced expression of activation molecules (CD39, CD69, and HLA-DR) and PD-1 inhibitory receptor on CD38^+^ T cells only in the patient cohort (**Fig. S8, B and C**).

We next evaluated the association of SARS-CoV-2-specific CD8^+^ T cells presence in the patient cohort related to their requirement for hospital care. No overall difference in the total number of recognized SARS-CoV-2-derived epitopes was observed between severely diseased patients requiring hospitalization (n=11 individuals) and patients with mild symptoms not requiring hospital care (outpatient; n = 7 individuals) (**Fig. 4C**). For phenotype characterization, 23 additional patient samples (total n=41 patients; Hospitalized n=21, Outpatients n=20) were analyzed using a patient HLA-matching pMHC multimer library combined with 13-parameter antibody panel, similar to the initial 18 patients but without resolving individual epitope specificities. Based on this extended cohort, a significantly higher frequency of SARS-CoV-2-specific CD8^+^ T cells was observed in the hospitalized patients compared to outpatient samples (**Fig. 4D**). Furthermore, a significant increase in the fraction of such cells expressing CD38, CD39, HLA-DR and PD-1 was observed in the hospitalized patients (**Fig. 4E**). By measuring co-expression of immune activation markers, CD38 together with CD39, PD-1, and HLA-DR, a strong elevation in T cells expressing these combinations of activation markers was observed among the hospitalized patients (**Fig. 4F**). Taken together, the increased frequency and activation signature suggests a role for SARS-CoV-2-specific CD8^+^ T cells in severe COVID-19 disease.

We also examined the phenotype of CD8^+^ T cells specific to the two most immunodominant epitopes TTDPSFLRGY and SPRWYFYYL with respect to disease severity (in eight patients; four hospitalized and four outpatients) using conventional pMHC tetramer-based evaluation of individual T cell specificities. Hospitalized patients displayed increased PD-1 expression compared to the same T cell populations in the outpatients (**Fig. S9A**). Furthermore, a higher frequency of T cells reactive to these two SARS-CoV-2 immunodominant epitopes was observed in the hospitalized patients, but the functional evaluation upon peptide stimulation revealed that only a sub-fraction of these high-frequency T cells where responsive to antigen exposure (**Fig. 4G****, Fig. S9B**). These data together with increased PD-1 expression suggests a functional impairment or selective inhibition of these high-frequent T cell populations, as observed by a recent study (*5*).

### A fraction of SARS-CoV-2 epitopes share sequence homology with widely circulating common cold coronaviruses

**Fig. 5:**
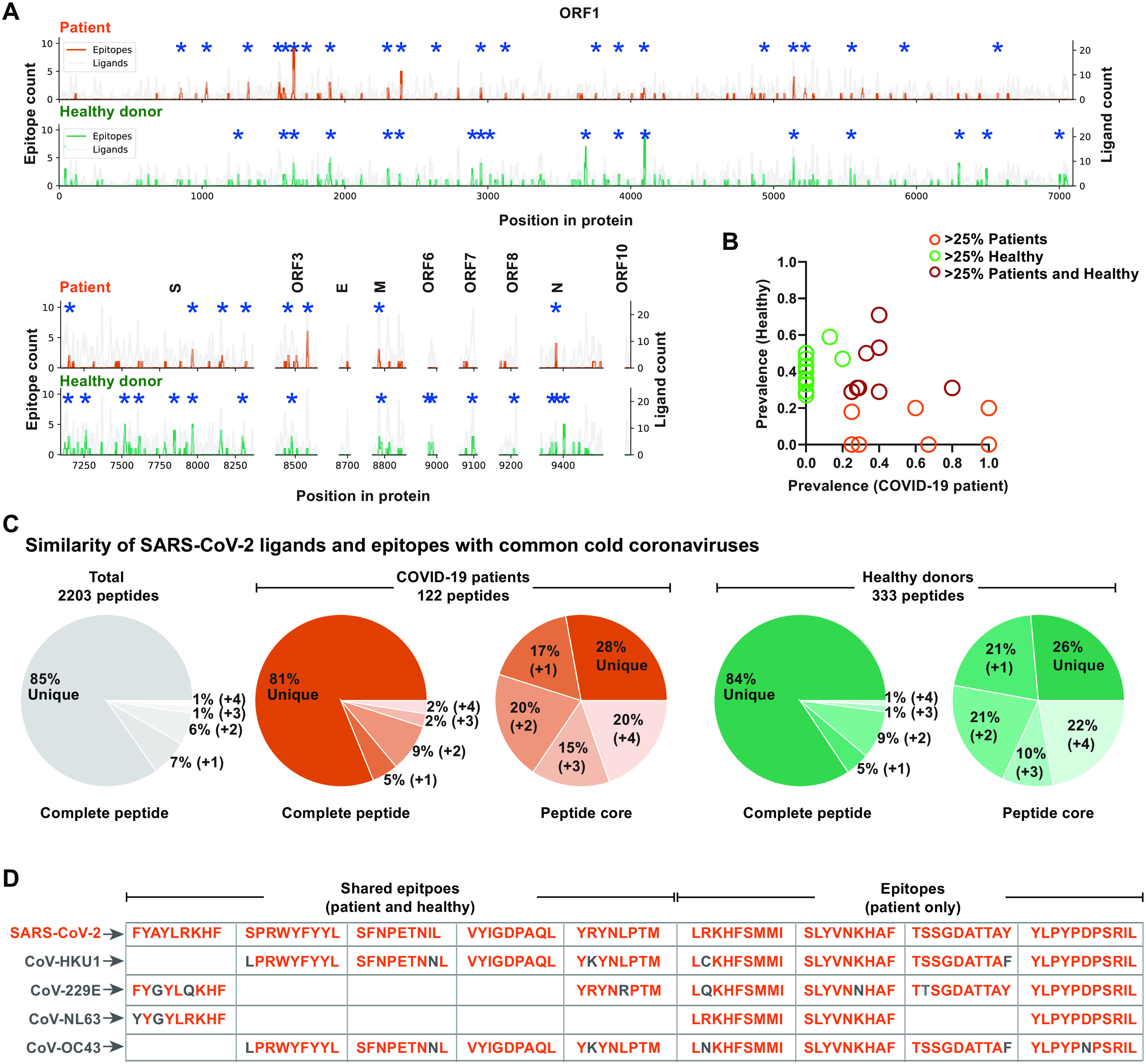
A fraction of SARS-CoV-2 epitopes shares sequence homology with widely circulating common cold coronaviruses. (**A**) SARS-CoV-2 T cell immunogenicity map across the viral proteome comparing the distribution of identified SARS-CoV-2 epitopes (patient cohort, orange line; n=16 patients) with the total analyzed peptides (gray line). The height of a peak indicates the number of ligands (right y-axis) analyzed in a particular region and the number of identified epitopes (left y-axis). The lower panel similarly maps epitopes and ligands from healthy donors (green line, n=31 individuals). Positions significantly enriched (p-value <0.05) with epitopes compared to the number of tested ligands are marked with asterisk. (**B**) T cell epitopes selected based on their immunodominant characteristics either in the patient (orange) or healthy donor (green) cohort, or represented in both (red) is evaluated for their T cell recognition prevalence in both cohorts. (**C**) Sequence similarity SARS-CoV-2 peptides with the other four common cold coronaviruses (HCoV): HCoV-HKU1, HCoV-NL63, and HCoV-229E. The gray pie chart indicates the sequence similarity of total predicted peptides from SARS-CoV-2 with any one (+1), two (+2), three (+3), or all four (+4) HCoV peptides with a variation limit of up to two amino acids within the full-length peptide. The colored pie chart shows a similar analysis for epitopes detected in the patient (n=16) or healthy donor cohort (combined analysis of HD-1 and HD-2, n=31) for full-length peptide and peptide core. (**D**) Examples of sequence homology for shared (between patient and healthy donors) and patient-specific T cell epitopes with one or more HCoV peptide sequence. Non-matching amino acids are shown in gray.

Pre-existing T cell immunity, in the context of SARS-CoV-2-reactive T cells in unexposed healthy individuals, has been reported by several studies (*13*,*15*, *17*, *19*), and it has been hypothesized that this is due to the shared sequence homology between the SARS-CoV-2 genome and other common cold coronaviruses (HCoV-OC43, HCoV-HKU1, HCoV-NL63, and HCoV-229E). Having evaluated the full spectrum of minimal epitopes for T cell recognition, we sought to evaluate the sequence homology at the peptide level, and its association with the SARS-CoV-2 T cell reactivity we observed in healthy donors. First, we searched for immunogenic hot-spots across the full SARS-CoV-2 proteome by comparing the number of identified epitopes (in the patient cohort) to the total number of predicted peptides in any given region of the proteins. Generally, the epitopes were spread over the full length of the protein sequences, while clustering in minor groups throughout all regions of the viral proteome (**Fig. 5A**). Regions indicated by asterisk demonstrate significant enrichment of T cell recognition relative to the number of MHC-I-binding peptides in a given region. Both the C- and N-terminal regions of the ORF1 seem to hold fewer T cell epitopes compared to the rest of this protein. When similarly mapping the T cell recognition of SARS-CoV-2-derived peptides observed in healthy donors, we detected a comparable spread of T cell recognition in the healthy donor cohort. Interestingly, most T cell epitope clusters in the patient cohort coincide with T cell recognition in the healthy donor cohort. The few regions that distinguish the T cell recognition observed in healthy donors from that observed in patients include: the C- and N-terminal regions of ORF1; parts of the N; and in general, a higher level of T cell recognition to S. In these regions, T cell recognition in healthy donors exceeded the observation from COVID-19 patients (**Fig. 5A**). Interestingly, when evaluating the prevalence of T cell recognition for the epitopes identified in >25% of the patient (**Fig. 2A****, Table S9**) or the healthy donor cohort (**Fig. S3C, Table S9**), we observed that most of these T cell responses frequently observed in COVID-19 patients are also detected in healthy donors, while a large fraction of epitopes dominating in healthy individuals were not detected in our patient cohort (**Fig. 5B**). However, several SARS-CoV-2 reactivities that were identified only in the healthy donors in our study were shown to be present in COVID-19 patients analyzed by other studies (**Table S9**), which strongly points to a substantial degree of cross-recognition to SARS-CoV-2 from pre-existing T cell populations, and that such populations might drive the further expansion of T cell responses to SARS-CoV-2 infection.

To further elucidate the potential origin of such a cross-reactive T cell population in the healthy donor cohort, we next evaluated the sequence homology of SARS-CoV-2 MHC-I-binding peptides with the four common cold coronaviruses: HCoV-HKU1, HCoV-NL63, and HCoV-229E. With a variation limit of up to two amino acids in each peptide sequence, 15% of the total predicted peptides showed sequence similarity with one or more HCoV peptide sequence (**Fig. 5C****, grey pie**). Among the T cell-recognized peptides, in both the patient and healthy donor cohorts, this fraction was comparable with 19% and 16%, respectively, of T cell recognized peptides sharing sequence homology with one or more HCoV (**Fig. 5C**). As an alternative approach, the similarities were calculated by kernel method for amino acid sequences using BLOSUM62, indicating comparable sequence similarity of peptides recognized by T cells and those not recognized in reference to HCoV. However, interestingly, peptides of lowest similarity to HCoV were not recognized by T cells in the patient cohort (**Fig. S10**).

As T cell cross-recognition can often be driven by a few key interaction points, predominantly in the core of the peptide sequence (i.e., position 38) (*28*, *29*), we restricted the sequence similarity to the core of the peptide that would be most likely to interact with the TCR (*30*). Based on the protein-core only, up to 74% of all the identified epitopes showed sequence homology to HCoV (one or more) (**Fig. 5C**), suggesting these common cold viruses as a potential source of the observed low-avidity interactions in healthy donors. Further, when evaluating peptides frequently recognized by T cells in both COVID-19 patients and healthy individuals, we find evidence of substantial homology, as exemplified with the peptide sequences listed in **Fig. 5D**. However, similar sequence homology is observed for the peptide sequences that are recognized only in the patient cohort (**Fig. 5D**). Thus, at present, our data points to substantial T cell cross-recognition being involved in shaping the T cell response to SARS-CoV-2 in COVID-19 patients; however, we find no specific enrichment of T cell recognition to peptide sequences with large sequence homology compared to the total peptide library being evaluated, and the responses identified exclusively in the patient samples are not more specific to SARS-CoV-2 compared to those recognized in both cohorts. Interestingly, ORF1 displayed the highest T cell recognition immunogenicity and also the highest sequence identity to HCoV (40%, as oppose to 22-34% for all other SARS-CoV-2 proteins, calculated using direct sequence alignment). Future studies seeking to fully understand the role and origin of the underlying T cell cross-recognition will likely require an in-depth evaluation of pre- and post-infection samples.

## DISCUSSION

Several studies using overlapping peptide pools spanning different region of SARS-CoV-2 viral proteins have shown a broad range of T cell activation in convalescent COVID-19 patients (*8*, *9*, *11*, *14*, *15*, *31*,*35*). Our work now provides a detailed characterization of minimal epitopes derived from the complete SARS-CoV-2 genome for their CD8^+^ T cell immunogenicity, immunodominance, and functional and phenotypical characteristics in COVID-19 patients and healthy donors. We identified CD8^+^ T cell responses to 122 epitopes in 18 COVID-19 patients after screening for T cell recognition based on 3141 peptides derived from the full SARS-CoV-2 genome and selected based on their predicted HLA-binding capacity. Of these, a few immunodominant T cell epitopes were recognized in most of the patients. Strikingly, both dominant and subdominant T cell epitopes were cross-recognized by low-level pre-existing T cell populations in SARS-CoV-2 unexposed healthy individuals. We have observed that the SARS-CoV-2 dominant epitopes mount very strong T cell responses, with up to 27% of all CD8^+^ lymphocytes recognizing a single epitope (two overlapping peptides with same peptide core).

Initial analysis of SARS-CoV-2 unexposed individuals revealed substantial presence of CD4^+^ and CD8^+^ T cells cross-reactive to SARS-COV-2 peptides (*11*, *15*, *17*, *36****39*). Longitudinal analysis of cross-reactive and induced CD8^+^ T cells before and after SARS-CoV-2 infection has been followed in individual cases (*39*), but the role of pre-existing T cells in overall immune response and disease outcome is not yet established. Using genome-wide screen of expanded T cells, a recent study reported cross-reactivity to SARS-CoV epitopes in COVID-19 patients but not to other commonly circulating coronaviruses (*40*). Our ex vivo evaluation of all 3141 SARS-CoV-2-derived minimal epitopes in two healthy cohorts (COVID-19 unexposed and high-risk) shows extensive but low-frequency and low-avidity interaction with CD8^+^ T cells. Pre-existing immunity based on cross-reactive T cells can influence how the immune system reacts upon viral exposure, including through faster expansion of pre-existing memory cells upon initial exposure to viral infection. A similar outcome and benefit of pre-existing T cell immunity have been shown in the case of flu pandemic virus H1N1 (*41*, *42*). However, active stimulation of cross-reactive T cells could also lead to exhaustion of rapidly expanded T cells, similar to the higher PD-1 expression and reduced cytokine secretion of the SARS-CoV-2 immunodominant T cells observed by us and others (*5*, *43*, *44*). Additionally, hyperactivation of pre-existing T cells could contribute to short- and long-term disease severity via inflammation and autoimmunity, as increased production of IFN- by CD4^+^ and CD8^+^ T cells has been observed in severe COVID-19 patients (*45*). Furthermore, it has been reported (*46*) that SARS-CoV-2 infection can be a triggering factor for autoimmune reactions and severe pneumonia with sepsis leading to acute respiratory distress syndrome (ARDS), bone-marrow affection with pancytopenia and organ-specific autoimmunity (*47****49*). Importantly, pre-existing T cell immunity can influence vaccination outcomes, as they may induce a faster, but possibly selective immune response. The ORF1 protein regions are highly conserved within coronaviruses (*50*), and show the highest HCoV identity among SARS-CoV-2 proteins; and most of the immunodominant epitopes that we have identified belong to the ORF1 region. Thus, a detailed evaluation of these T cell epitopes could be of value in vaccine design.

Most vaccine development efforts are currently focusing on mounting antibody responses to the spike protein, with limited focus on T cell immunity. This is due to the receptor binding domain (RBD) being the main target for neutralizing antibodies produced by B cells (*51*). However, several studies have pointed out relatively low antibody titers in COVID-19 recovered patients (*3*, *52****54*). In conditions, where antibody titers cannot sufficiently protect against infections T cell immunity may sustain the antibody responses and provide a direct source of T cells for clearing virus-infected cells. For the involvement of T cell immunity in vaccine development, our data suggest that the inclusion of other virus proteins, such as ORF1 or 3, might be highly relevant. For now, the role of antibody- and T cell-mediated immune response post-natural infection or post-vaccination is not yet resolved and requires extensive longitudinal analysis comparing antibody and T cell kinetics to determine synergistic or specific effect in long-term disease protection.

T cell recognition of SARS-CoV-2-derived peptides in both COVID-19 patients and healthy donors has prompted us to understand the role of T cell cross-reactivity in controlling infections. In recent years, technology improvements in TCR characterization have allowed us to interrogate the TCR-pMHC interaction from a structural approach, while obtaining experimental information related to the peptide amino acids that are crucial to T cell recognition (*55****60*). Such efforts have taught us that T cell cross-recognition is very difficult to predict, without knowing the precise interaction required for the given TCR, as even T cell epitopes with as low as 40% sequence homology can be recognized by a given TCR (*30*). Therefore, the underlining source of T cell cross-reactivity might arise from a larger set of epitopes within the HCoV viruses, including sequences with larger variation than those evaluated here (i.e., maximum of two amino acid variants per peptide sequence/peptide core).

While the T cell recognition itself was largely overlapping in identity between patients and healthy donors, the magnitude of the T cell responses and, in particular, the phenotype of SARS-CoV-2-specific T cells was substantially different. We detected a strong activation profile of SARS-CoV-2-specific T cells only in COVID-19 patients, and this strong activation signature (high expression of CD38, CD39, PD1, HLA-DR) was further enhanced in patients requiring hospitalization. Such highly activated T cell responses should facilitate viral clearance, and hence our data further support the notion that some severely affected patients might suffer from hyperactivation of their T cell compartment as a consequence of their primary viral infection, which may even be cleared. Additional signs of functional impairment were observed and cytokine secretion upon antigen stimulation was incomplete for the high-frequent populations of SARS-CoV-2 specific T cells.

A limitation of current study relates to the lack of information related to the precise date of infection. This may differ by up to 1 week, as symptoms and hence diagnosis can be delayed. Consequently, differences in T cell mobilization and/or activation may be observed as a function of time, which is not controlled in the present study. However, a measurement of symptoms prior first positive SARS-CoV-2 test, indicates that samples were collected at approximately the same time relative to symptom onset in the two groups of patients, except for 3 patients from the ICU included later after infection. Additionally, although our T cell screening strategy allows for high-throughput epitope mapping, determination of individual responses can only be estimated following the barcode deconvolution strategy, in relation to the pool of pMHC multimer positive T cells upon sorting. For the healthy donor population, the separation was insufficient to precisely determine the frequency of this T cell population, while for the patient cohort, both measurements demonstrated strong correlation with measurements of the individual responses using conventional pMHC tetramers.

Taken together, COVID-19 disease drives substantial T cell activation, with T cell recognition of a large number of SARS-CoV-2-derived peptides. There is also considerable T cell recognition of such peptides in healthy donors, arguing for cross-recognition, potentially from T cells raised against other coronaviruses. Importantly, the activation profile clearly distinguish patients from healthy individuals. Interestingly, patients that required hospitalization for COVID-19 demonstrated a significantly higher frequency of SARS-CoV-2-specific T cells and with a more activated phenotype compared to patients with milder disease. The data presented here support a role for T cell recognition in COVID-19 and hypothesize that such T cells are associated with COVID-19 disease severity. Pre-existing T cell immunity likely influences the immune response to SARS-CoV-2, which could be leveraged to fight novel infections.

## MATERIALS AND METHODS

### Study design

This study aimed to identify a full repertoire of CD8^+^ T cell-mediated immune response to SARS-CoV-2 infection. For a comprehensive evaluation, we determined potential T cell epitopes within the complete SARS-CoV-2 genome and analyzed the resulting 3141 peptides for their T cell recognition, immunodominance, breadth of the T cell response, functional and phenotype of reactive T cells, and contribution in COVID-19 disease severity. We used a DNA-barcode based MHC multimer T cell detection technology in combination with a 13-parameter flow cytometry phenotyping panel for T cell identification in PBMCs in a cohort of 18 COVID-19 patients (composed of severe and mild disease) and compared with T cell recognition in two healthy donor cohorts (18 COVID-19 unexposed individuals and 20 high-risk healthcare staff). To understand association of SARS-CoV-2 specific T cells in disease severity we included an additional 23 patients for T cell phenotype analysis.

### Clinical samples

Approval for the study design and sample collection was obtained from the Committee on Health Research Ethics in the Capital Region of Denmark. All included patients and health care employees gave their informed written consent for inclusion. PBMC samples from 18 SARS-CoV-2-infected patients were used in this study. Blood samples were collected as close as possible to the first COVID-19 positive test. The patient cohort included samples from individuals with severe symptoms who required hospital care (hospitalized; n = 11) and patients with mild symptoms not requiring hospital care (outpatient; n = 7). For hospitalized patients, we collected full data from the medical record regarding disease course, age, gender, travel history, performance status, symptoms, comorbidity, medications, laboratory findings, diagnostic imaging, treatment, need of oxygen, need for intensive care, and an overall estimate of disease severity (**Table S2**). For outpatients, we used a questionnaire to collect data on comorbidity, travel history, medications, and performance status.

SARS-CoV-2 infection was diagnosed by one of the four platforms; BGI (BGI Covid-19 RT-PCR kit), PantherFusion (Hologic), Roche Flow (Roche MagNA Pure 96, Roche LightCycler 480 II real-time PCR), and Qiaflow (QIAsymphony or RotorGene, Qiagen). In the last three platforms, LightMix Modular SARS-CoV (COVID-19) E-gene (# 53-0776-96) has been used. The diversity of platforms used were due to supply issues. All platforms were validated using validation kits and panels from the Statens Serum Institute (SSI), Denmark. Most patients had more than one positive test for COVID-19. Swabs, sputum, and tracheal secretion were used depending on the setting.

Patients were attempted for inclusion soon after diagnosis. The samples were collected within 2 weeks from COVID-19 diagnosis (except for three patients who were at intensive care post diagnosis). The average number of days with symptoms before sample collection matches closely in the two patient cohorts (10.85 days for hospitalized group and 10.45 days for outpatient group) (**Table S2)**; however, it was not possible to determine the exact date of infection.

For the pre-COVID-19 healthy donor cohort (n = 18), we used samples collected prior to October 2019 and obtained from the central blood bank, Rigshospitalet, Copenhagen, in an anonymized form. Additionally, we included 20 health care employees from Herlev Hospital during the COVID-19 pandemic, who were at high risk of SARS-CoV-2 infection but not detected to be positive, as a cohort to follow immune responses in a potentially exposed population.

PBMCs from all three cohorts were isolated immediately after sampling using Ficoll-Paque PLUS (GE Healthcare) density gradient centrifugation and were cryopreserved thereafter at a density of 220 10^6^ cells/mL.

### SARS-CoV-2 peptide selection

Potential HLA class I binding peptides were predicted from the complete set of 811mer peptides contained within the Wuhan seafood market pneumonia virus isolate Wuhan-Hu-1 (GenBank ID MN908947.3) to a set of ten prevalent and functionally diverse HLA molecules (HLA-A01:01, HLA-A02:01, HLA-A03:01, HLA-A24:02, HLA-B07:02, HLA-B08:01, HLA-B15:01 HLA-C06:02, HLA-C07:01, HLA-C07:02) using a preliminary version of NetMHCpan 4.1(http://www.cbs.dtu.dk/services/NetMHCpan/index_v0.php)[PMID: 32406916]. For peptides predicted from ORF1 protein, a percentile rank binding threshold of 0.5% was used, and for peptides derived from all other proteins, a threshold of 1% was used. Altogether, 2203 peptides were selected, binding to one or more HLA molecules, generating 3141 peptide-HLA pairs for experimental evaluation (**Table S1**). All peptides were custom synthesized by Pepscan Presto BV, Lelystad, The Netherlands. Peptide synthesis was done at a 2 mol scale with UV and mass spec quality control analysis for 5% random peptides with estimated purity of 70-92% by the supplier.

### MHC class I monomer production

All ten MHC-I monomer types were produced using methods previously described (*61*). Briefly, MHC-I heavy chain and human 2-microglobulin (h2m) were expressed in *Escherichia coli* using pET series expression plasmids. Soluble denatured proteins of the heavy chain and h2m were harvested using inclusion body preparation. The folding of these molecules was initiated in the presence of UV labile HLA specific peptide ligands (*62*). HLA-A02:01 and A24:02 molecules were folded and purified empty, as described previously (*63*). Folded MHC-I molecules were biotinylated using the BirA biotin-protein ligase standard reaction kit (Avidity, LLC- Aurora, Colorado), and MHC-I monomers were purified using size exclusion chromatography (HPLC, Waters Corporation, USA). All MHC-I folded monomers were quality controlled for their concentration, UV degradation, and biotinylation efficiency, and stored at -80C until further use.

### DNA-barcoded multimer library preparation

The DNA-barcoded multimer library was prepared using the method developed by Bentzen *et al*. (*25*). Unique barcodes were generated by combining different A and B oligos, with each barcode representing a 5 biotinylated unique DNA sequence. These barcodes were attached to phycoerythrin (PE) or allophycocyanin (APC) and streptavidin-conjugated dextran (Fina BioSolutions, Rockville, MD, USA) by incubating them at 4C for 30 min to generate a DNA-barcode-dextran library of 1325 unique barcode specificities. SARS-CoV-2 pMHC libraries were generated by incubating 200 M peptide of each peptide with 100 g/mL of respective MHC molecules for 1 hour using UV-mediated peptide exchange (HLA-A01:01, A03:01, B07:02, B08:01, B15:01, C06:02, C07:01, and C07:02) or direct binding to empty MHC molecules (HLA-A02:01 and A24:02). HLA-specific DNA-barcoded multimer libraries were then generated by incubating pMHC monomers to their corresponding DNA barcode-labeled dextrans at 4C for 30 min, thus providing a DNA barcode-labeled peptide-MHC (pMHC) multimer specifically to probe respective T cell population. A similar process was followed to generate DNA-barcoded pMHC multimers for CEF epitopes (HLA-A and HLA-B) using APC- and streptavidin-conjugated dextran attached with unique barcodes.

### T cell staining with DNA-barcoded pMHC multimers and phenotype panel

All COVID-19 patient and healthy donor samples were HLA genotyped for HLA-A, B, and C loci (IMGM Laboratories GmbH, Germany, next-generation sequencing) (**Table S10**). Patient and healthy donor HLA-matching SARS-CoV-2 and CEF pMHC multimer libraries were pooled (as described previously (*25*)) and incubated with 510 10^6^ PBMCs (thawed and washed twice in RPMI + 10% FCS, and washed once in barcode cytometry buffer) for 15 min at 37C at a final volume of 60 L. Cells were then mixed with 40 L of phenotype panel containing surface marker antibodies (**Table S8**) and a dead cell marker (LIVE/DEAD Fixable Near-IR; Invitrogen L10119) (final dilution 1/1000), and incubated at 4C for 30 min. Cells were washed twice with barcode cytometry buffer and fixed in 1% PFA.

Cells fixed after staining with pMHC-multimers were acquired on a FACSAria flow cytometer instrument (AriaFusion, Becton Dickinson) and gated by the FACSDiva acquisition program (Becton Dickinson), and all the PE-positive (SARS-CoV-2 multimer binding) and APC-positive (CEF multimer binding) cells of CD8^+^ gate were sorted into pre-saturated tubes (2% BSA, 100 l barcode cytometry buffer) (**Fig. S11A**). Sorted cells belonging to each sample were then subjected to PCR amplification of its associated DNA barcode(s). Cells were centrifuged for 10 min at 5000 g, and the supernatant was discarded with minimal residual volume. The remaining pellet was used as the PCR template for each of the sorted samples and amplified using the Taq PCR Master Mix Kit (Qiagen, 201443) and sample-specific forward primer (serving as sample identifier) A-key36. PCR-amplified DNA barcodes were purified using the QIAquick PCR Purification kit (Qiagen, 28104) and sequenced at PrimBio (USA) using the Ion Torrent PGM 314 or 316 chip (Life Technologies).

### DNA-barcode sequence analysis and identification of pMHC specificities

To process the sequencing data and automatically identify the barcode sequences, we designed a specific software package, Barracoda (https://services.healthtech.dtu.dk/service.php?Barracoda-1.8). This software tool identifies the barcodes used in a given experiment, assigns sample ID and pMHC specificity to each barcode, and calculates the total number of reads and clonally reduced reads for each pMHC-associated DNA barcode. Furthermore, it includes statistical processing of the data. Details are given in Bentzen *et al*. (*25*). The analysis of barcode enrichment was based on methods designed for the analysis of RNA-seq data and was implemented in the R package edgeR. Fold changes in read counts mapped to a given sample relative to mean read counts mapped to triplicate baseline samples were estimated using normalization factors determined by the trimmed mean of M-values. P-values were calculated by comparing each experiment individually to the mean baseline sample reads using a negative binomial distribution with a fixed dispersion parameter set to 0.1 (*25*). False-discovery rates (FDRs) were estimated using the Benjamini-Hochberg method. Specific barcodes with an FDR < 0.1% were defined as significant, determining T cell recognition in the given sample. At least 1/1000 reads associated with a given DNA barcode relative to the total number of DNA barcode reads in that given sample was set as the threshold to avoid false-positive detection of T cell populations due to the low number of reads in the baseline samples. T cell frequency associated with each significantly enriched barcode was measured based on the % read count of the associated barcode out of the total % multimer-positive CD8^+^ T cells population in patient samples. In healthy donors, the T cell recognition were identified based on barcode enrichment analysis, same as in patient samples, however, a frequency estimate of the corresponding T cell populations was not determined for significant responses identified in healthy donors due to insufficient separation of multimer positive cells. To exclude potential pMHC elements binding to T cells in a non-specific fashion, non-HLA-matching healthy donor material was included as a negative control. Any T cell recognition determined in this samples was subtracted from the full data set.

### T cell expansion and combinatorial tetramer staining

PBMCs from healthy donors were expanded *in-vitro* using pMHC-dextran complexes conjugated with SARS-CoV-2-derived peptides and cytokines (IL-2 and IL-21) for 2 weeks either with single pMHC specificity or with a pool of up to ten pMHC specificities. PBMCs were expanded for 2 weeks in X-vivo media (Lonza, BE02-060Q) supplemented with 5% human serum (Gibco, 1027-106). Expanded cells were used to measure peptide-specific T cell activation or stained using pMHC tetramers to detect T cells recognizing SARS-CoV-2 epitopes.

*In-vitro* expanded healthy donor PBMCs were examined for SARS-CoV-2 reactive T cells using combinatorial tetramer staining (*64*). Individual HLA-restricted pMHC complexes were generated using direct peptide loading (HLA-A02:01 and A24:02) or UV-mediated peptide exchange (all other HLAs) as described above and conjugated with fluorophore-labeled streptavidin molecules. For 100 L pMHC monomers, 9.02 L (0.2 mg/mL stock, SA-PE-CF594 (Streptavidin - Phycoerythrin/CF594; BD Biosciences 562318), SA-APC (Biolegend 405207) or 18.04 L (0.1 mg/mL stock, SA-BUV395 (Brilliant Ultraviolet 395; BD 564176), SA-BV421 (Brilliant Violet 421; BD 563259), and SA-BV605 (Brilliant Violet 605; BD 563260) of streptavidin conjugates were added and incubated for 30 min at 4C, followed by addition of D-biotin (Sigma) at 25 M final concentration to block any free binding site. pMHC tetramers for each specificity were generated in two colors by incubating pMHC monomers and mixed in a 1:1 ratio before staining the cells. Expanded cells were stained with 1 L of pooled pMHC multimers per specificity (in combinatorial encoding) by incubating 15 10^6^ cells for 15 min at 37C in 80 L total volume. Cells were then mixed with 20 L antibody staining solution CD8-BV480 (BD B566121) (final dilution 1/50), dump channel antibodies (CD4-FITC (BD 345768) (final dilution 1/80), CD14-FITC (BD 345784) (final dilution 1/32), CD19-FITC (BD 345776) (final dilution 1/16), CD40-FITC (Serotech MCA1590F) (final dilution 1/40), CD16-FITC (BD 335035) (final dilution 1/64)) and a dead cell marker (LIVE/DEAD Fixable Near-IR; Invitrogen L10119) (final dilution 1/1000) and incubated for 30 min at 4C. Cells were then washed twice in FACS buffer (PBS+2% FCS) and acquired on a flow cytometer (Fortessa, Becton Dickinson). Data were analyzed using FlowJo analysis software.

### T cell functional analysis

For functional evaluation of T cells from PBMCs of COVID-19 patients or PBMCs expanded from healthy donors, 12 10^6^ cells were incubated with 1 M of SARS-CoV-2 minimal epitope or pool of up to ten epitopes (1 M/peptide) for 9 hours at 37C in the presence of protein transport inhibitor (GolgiPlug; BD Biosciences, 555029; final dilution 1/1000). Functional activation of T cells was measured using intracellular cytokines IFN- (BD Bioscience, 341117; final dilution 1/20) and TNF- (Biolegend, 502930; final dilution 1/20). Cells incubated with Leukocyte Activation Cocktail (BD Biosciences, 550583; final dilution 1/500) were used as a positive control, and HLA-specific irrelevant peptides were used as negative controls. Surface marker antibodies CD3-FITC (BD Biosciences 345764 (final dilution 1/20)), CD4-BUV395 (BD Biosciences 742738 (final dilution 1/300), CD8-BV480 (BD Biosciences B566121 (final dilution 1/50)), and dead cell marker (LIVE/DEAD Fixable Near-IR; Invitrogen L10119) (final dilution 1/1000)) were used to identify CD8^+^ T cells producing intracellular cytokines (Gating strategy, **Fig. S5A**).

### Flow cytometry analysis

For phenotype analysis, all samples were analyzed using FlowJo data analysis software (FlowJO LLC). Frequencies of specific cell populations were calculated according to the gating strategy shown in **Fig. S11B**. For combinatorial tetramers staining, T cell binding to specific-pMHC tetramers was identified using the gating plan described in the original study (*65*). For UMAP analysis (*66*), FCS files of samples from the patient and healthy cohorts were concatenated (160,000 total cells), downsampled (FlowJo plugin), and visualized using UMAP (Version 2.2, FLowJo plugin) analysis based on the selected markers; CD3, CD4, CD8, CD38, CD39, CD69, CD137, HLA-DR, PD-1, CCR7, CD45RA, CD27, and CD57.

### Sequence homology analyses

To evaluate the homology between SARS-CoV-2 and HCoV, both epitopes (peptides recognized by T cells) and ligands (peptide not recognized by T cells) were mapped to their respective source protein from the SARS-CoV-2 proteome. Enrichment analysis of the epitopes in the given region of the proteins was based on testing whether the count of observed epitopes exceeded what we expected from the number of ligands tested at each position. Epitopes were considered successes and the count of ligands were regarded as the number of trials in a binomial test. The probability of success was derived from the average ratio of epitope to ligand per position across each protein. The test was one-sided with a significance level at 0.05.

The similarity of SARS-CoV-2 ligands and epitopes from both patient and healthy donor cohorts to a set of human common cold corona viruses (HCoV-HKU1, HCoV-229E, HCoV-NL63, HCoV-OC43) was tested using two methods. The first approach utilized a kernel method for amino acid sequences using BLOSUM62 (*67*). The second approach was a simple string search allowing up to two mismatches. Based on the second approach each epitope was categorized by how many, if any, of the common cold viruses it would match with. Both methods were applied to the full peptide length and to the peptide core.

### Data processing and statistics

T cell recognition was determined based on the DNA-barcoded pMHC multimer analysis and evaluated through barracoda (see above). The data was plotted using python 3.7.4. For all plots, peptide sequences with no significant enrichments are shown as gray dots and all peptide with a negative enrichment are set to LogFc equal zero (**Fig. 1, D, E and G****;** **Fig. 3, A and B****; Fig. S2**). Box plots for data quantification and visualization were generated, and their related statistical analyses were performed using GraphPad Prism (GraphPad Software Inc.) (**Fig. 3C****;** **Fig. 4A-F****; Fig. S1, A and B, Fig. S7B, Fig. S8, B and C, Fig. S9A**) or R studio (**Fig. S10**). For unpaired comparisons Mann-Whitney test was applied, and to compare more than two groups One-Way ANOVA (Kruskal-Wallis) test was performed using GraphPad Prism. All p values are indicated in figure legends. Flow Cytometry data were analyzed using FlowJo (version 10). Immunogenicity scores (**Fig. 1, F and H****; Fig. S3**) were calculated (as %) by dividing total identified T cell reactivity associated with a HLA or protein with the total number of specificities analyzed in a given cohort (number of peptides multiplied by number of patient with a given HLA). Staining index (**Fig. 3C**) was calculated as; ((mean fluorescence intensity (MFI) of multimer^+^ cells MFI of multimer^-^ cells)/2 standard deviation (SD) of multimer^-^ cells)). MFI of multimer^+^ and multimer^-^ CD8^+^ T cells and the SD of the multimer^-^ CD8^+^ T cells from FlowJo analysis for patient and healthy donor samples.

## Supplementary materials

immunology.sciencemag.org/cgi/content/full/6/58/eabf7550/DC1 **Fig. S1.** Details of SARS-CoV-2 derived peptides in relation to their HLA and patient-specific coverage and immunogenicity. **Fig. S2.** Examples of genome-wide screening for SARS-CoV-2-reactive T cells in individual COVID 19 patient samples. **Fig. S3.** Summary of SARS-CoV-2-specific T cell reactivity identified in healthy donors. **Fig. S4.** Representative flow cytometry plots of SARS-CoV-2-specific T cell multimer recognition in healthy donors. **Fig. S5.** Gating strategy and functional evaluation of SARS-CoV-2-reactive CD8^+^ T cells identified in healthy donors. **Fig. S6.** UMAP visualization comparing phenotype characteristics of SARS-CoV-2 peptide reactive T cells in the COVID 19 patient and healthy donor cohorts. **Fig. S7.** Memory phenotype of SARS-CoV-2-reactive CD8^+^ T cells. **Fig. S8.** Phenotype comparison of CEF- and SARS-CoV-2-specific T cell responses. **Fig. S9.** Phenotype and functional evaluation of the immunodominant epitopes in hospitalized and outpatients. **Fig. S10.** Sequence similarities of SARS-CoV-2 peptides with HCoV. **Fig. S11.** Gating strategy for sorting multimer^+^ CD8^+^ T cells and phenotype analysis. **Table S1.** List of SARS-CoV-2 peptides analyzed for T cell reactivity with their HLA rank score (Excel file). **Table S2.** COVID-19 patient information. **Table S3.** CEF epitope library (Excel file). **Table S4.** SARS-CoV-2 -specific T cell epitopes identified in COVID-19 patients (Excel file). **Table S5.** CEF-specific T cell responses identified in COVID-19 patients, in pre-COVID-19 healthy donors (HD-1) and in high-risk healthy donors (HD-2) (Excel file). **Table S6.** Functional analysis of SARS-CoV-2 antigen-specific T cells in COVID-19 patients. **Table S7.** SARS-CoV-2 -specific T cell epitopes identified in pre-COVID-19 healthy donors (HD-1) and high-risk healthy donors (HD-2) (Excel file). **Table S8.** Phenotype flow cytometry antibody panel. **Table S9.** SARS-CoV-2 T cell epitopes with comparison to previously reported epitopes. **Table S10.** HLA genotype data for patient and healthy donors (Excel file). **Table S11.** Raw data file (Excel file).
